# Evidence of HARKing in mouse behavioural tests of anxiety

**DOI:** 10.1098/rsos.231744

**Published:** 2024-08-21

**Authors:** Marianna Rosso, Adrian Herrera, Hanno Würbel, Bernhard Voelkl

**Affiliations:** ^1^ Animal Welfare Division, University of Bern, Länggassstrasse 120, Bern 3012, Switzerland

**Keywords:** HARKing, behavioural test, anxiety

## Abstract

Over the last decades, behavioural tests in animals, especially rodents, have been a standard screening method to determine the mechanisms of action and efficacy of psychopharmacological compounds. Yet, recently the reproducibility of some of these tests has been questioned. Based on a systematic review of the sensitivity of mouse behavioural tests to anxiolytic drugs, we analysed behavioural outcomes extracted from 206 studies testing the effect of diazepam in either the open-field test or the hole-board test. Surprisingly, we found that both the rationale given for using the test, whether to detect anxiolytic or sedative effects, and the predicted effect of diazepam, anxiolytic or sedative, strongly depended on the reported test results. The most likely explanation for such strong dependency is post hoc reasoning, also called hypothesizing after the results are known (HARKing). HARKing can invalidate study outcomes and hampers evidence synthesis by inflating effect sizes. It may also lead researchers into blind alleys, and waste animals, time and resources for inconclusive research.

## 1. Introduction

Anxiety disorders are among the most common mental health conditions needing new and better treatments. Development of anxiolytic drugs using animal models heavily relies on behavioural tests, most of which measure anxiolytic effects by increased exploratory activity. However, interpretation of such tests is ambiguous because reduced exploratory activity can reflect either anxiety or sedation by the compound. Recently, the validity of behavioural tests for anxiety in rodents has been questioned, as they often fail to replicate results with different anxiolytic compounds or different mouse models [1,2]. To investigate whether this critique is justified, we have previously conducted a systematic review on the sensitivity of a wide range of measures of common mouse behavioural tests of anxiety to detect anxiolytic drugs approved for treating anxiety in humans [3]. Across the 2476 behavioural outcomes analysed, we found considerable variation in both direction and magnitude of effects within the same test measure, compound and dosage. Although some variation in effect size between studies is expected, we found contradictory results. Notably, for two test measures—‘number of squares crossed’ in the open-field test and ‘number of head dips’ in the hole-board test—diazepam produced almost equal numbers of positive and negative effects despite comparable test conditions. To understand these contradicting results, we further analysed this pool of research papers by assessing the interpretation of the test results and its relation to the rationales given for performing these tests, and the predicted effects of the compound.

## 2. Methods

The study is based on data of a systematic review [3], which synthesized evidence for the effects of anxiolytic compounds on outcome measures in behavioural tests using mice. For the current analysis, we set additional inclusion criteria, by focusing on the most frequently tested compound (diazepam), two behavioural measures (number of squares crossed in the open-field test and number of head dips in the hole-board test) and a maximum dosage of 2 mg kg^−1^ diazepam to ensure that results were comparable. The resulting subset of data comprises 241 outcomes from 206 publications assessing the effect of diazepam, of which 151 outcomes were based on ‘number of squares crossed’ in the open-field test and 90 were based on ‘number of head dips’ in the hole-board test. As dosage is known as a critical variable affecting the effect of diazepam, we conducted also a post hoc analysis, where we restricted the dataset further by only including results where a dosage of exactly 1 mg kg^−1^ was administrated, resulting in a set of 90 outcomes for the open-field test and 50 outcomes for the hole-board test. A summary of this additional analysis is given in the electronic supplementary material.

For each paper, two independent reviewers extracted information regarding (i) the interpretation of the observed effect of diazepam, (ii) the test rationale, and (iii) the predicted effect of diazepam. This was done by searching for statements which would answer the following three questions. First, were the observed behavioural changes elicited by diazepam described as sedative or anxiolytic effects? Second, was the test measure according to the authors meant to measure anxiolytic or sedative effects? Third, was diazepam administered as a sedative drug or as an anxiolytic drug? While in some cases, the study authors explicitly addressed these questions, frequently the authors either did not elaborate on these questions or made vague or ambiguous statements (e.g. the test measure was used to ‘assess anxiety, locomotion and emotionality’). In those cases, we classified the authors’ stance as ’ambiguous’. For each test measure, the dataset included mean values, sample size and standard deviation for both treatment and control groups (electronic supplementary material, table S1).

**Figure 1.**
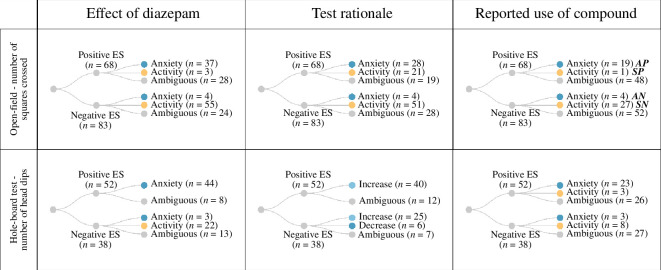
Distribution of outcomes across outcome measure, effect size (ES), item of analysis and reporting.

Assuming that predicted and observed effects should be independent for those researchers who openly report their results regardless of their prediction of the effect, we can make statements about the most likely scenario leading to the observed reporting patterns as follows. Administration of diazepam can have a positive effect on the outcome measure (increase in numbers of squares crossed or head dips) or a negative effect (decrease in number of squares crossed or head dips). Combining the observed effect of diazepam with the prediction of the researcher regarding the working of diazepam, which can be either stated as being sedative or anxiolytic, we arrive at a 2 × 2 matrix of four possible types of observed outcomes:


$$O=\left[\begin{array}{cc} SN & SP\\ AN & AP \end{array}\right],$$

where *SN* is the number of times a researcher reported a negative effect and declared that diazepam was used as sedative, *SP* is the number of times a researcher reported a positive effect and declared that diazepam was used as sedative, *AN* is the number of times a researcher reported a negative effect and declared that diazepam was used as anxiolytic and *AP* is the number of times a researcher reported a positive effect and declared that diazepam was used as anxiolytic (figure 1).

If we have a population of *n* researchers, each reporting one outcome, with *a* being the number of researchers expecting an anxiolytic effect, but openly reporting their result irrespectively of their expectation, *s* being the number of researchers expecting a sedative effect, but openly reporting their result irrespectively of their expectation, then the matrix


E=n P/k ,

gives the expected outcomes for given numbers of *a*, *s* and *n*, a reporting bias of *β* and observed proportions of positive ( φ ) and negative ( ω ) results. The scalar *k* is the grand sum of the matrix *P*, k=∑∑P , and


$$P=\left(\begin{array}{cc} \omega (\vartheta +\sigma ) & \sigma \varphi (1-\beta )\\ \alpha \omega (1-\beta ) & \varphi (\vartheta +\alpha ) \end{array}\right),$$

where $\alpha =\frac{a}{n},\;\sigma =\frac{s}{n}and\;\vartheta =\frac{n-a-s}{n}.$ The publication bias *β* is the proportion of researchers that would not publish a result contrary to their expectation. For each combination of *a* and *s* and a given publication bias, we can calculate the Frobenius *d* distance between *E* and *O* as


$$d\left(E,O\right)=\sqrt{Tr\left(D^{\prime }D\right)},$$

where D=E−O.

We can then consider the expected outcome matrix *E* with the combination of *a* and *s* resulting in the smallest distance ( $d_{min}$ ) to the observed outcome matrix *O* as the scenario which is most consistent with the observed patterns. As *α*, *σ* and *ϑ* are proportional to the number of researchers openly reporting observed effects and expecting an anxiolytic effect (*α*), openly reporting observed effects and expecting a sedative effect (*σ*) and researchers hypothesizing after results are known (HARKers*; ϑ*), we can provide these proportions and plot them against the strength of the publication bias (figure 3). The analysis of the stated rationale for the choice of measure can be done likewise. An annotated script for calculating these proportions is provided in the electronic supplementary material.

## 3. Results

**Figure 2.**
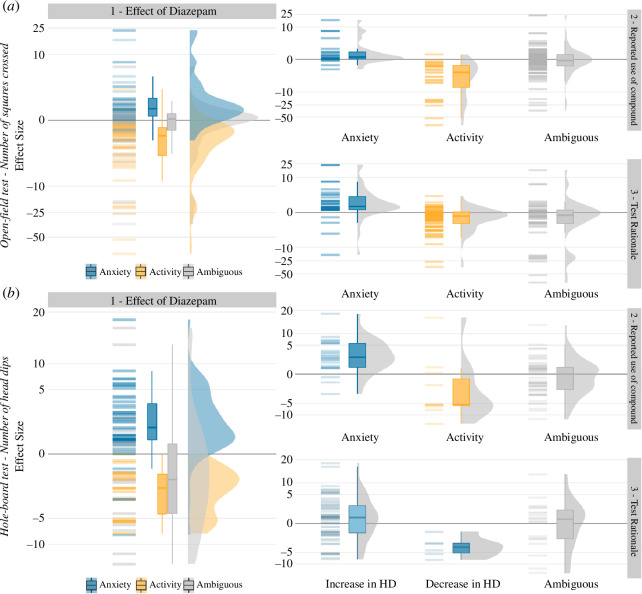
Relation between (1) effect of diazepam, (2) reported use of diazepam and (3) rationale for the use of the test measure and effect size, for the number of squares crossed in the open-field test (*a*) and number of head dips (HD) in the hole-board test (*b*). Horizontal bars, boxplots and violin plots indicate the distribution of the effect sizes as extracted from the publications.

In both tests, a decrease in activity consistently led researchers to conclude that diazepam had a sedative or depressant effect, while an increase in the activity was almost always interpreted as diazepam having an anxiolytic effect (figure 2). This result fits with the description of these tests by their inventors [4,5]. However, when it comes to the predicted effect of diazepam and the test rationale, we observed clear discrepancies in the study descriptions.

The reported rationale for the use of diazepam showed an unexpected bidirectional pattern. In 42 out of 46 cases where a positive effect size was detected and a prediction for the effect of diazepam was made (both tests combined), researchers described diazepam as a standard anxiolytic drug. However, when researchers found a negative effect size, diazepam was described as a standard sedative drug in 35 out of the 42 cases in which a prediction was made. In 153 cases, where the authors did not predict the effect of diazepam, or the explanation was ambiguous, both negative and positive effect sizes were reported approximately equally often (figures 1 and 2).

Similarly, the rationale provided for measuring ‘numbers of squares crossed’ in the open-field test was dependent on the study outcome. In 28 out of 49 cases where a positive effect was detected and a rationale was given, researchers presented the test measure as a tool to assess anxiety, while in 51 out of 55 studies where a negative effect was detected and a rationale was given, researchers described the same test measure as an assessment tool for locomotor activity.

In all studies, researchers used comparable dosages and usually the same method and route of administration. These factors are, therefore, unlikely to be responsible for the observed variation in the outcomes. If the observed variation in reported effect sizes is primarily due to a random sample variation, it should be independent of the researchers’ predictions about the effect of the compound or their rationale for using the test measure. This is, however, not the case. There are two possible explanations for these unexpected results: publication bias and HARKing. That is, such deviation from random variation could be explained, if researchers were more likely to publish results that matched their predictions (the file drawer problem, [6]) or if they formulated their hypotheses after the results had been known (HARKing, [7]).

Given these two alternative explanations, the question arises of how strong a tendency for HARKing or publication bias, or both, would have to be to explain the discrepancies in the test rationales and predicted effects discovered in the present meta-analysis.

**Figure 3.**
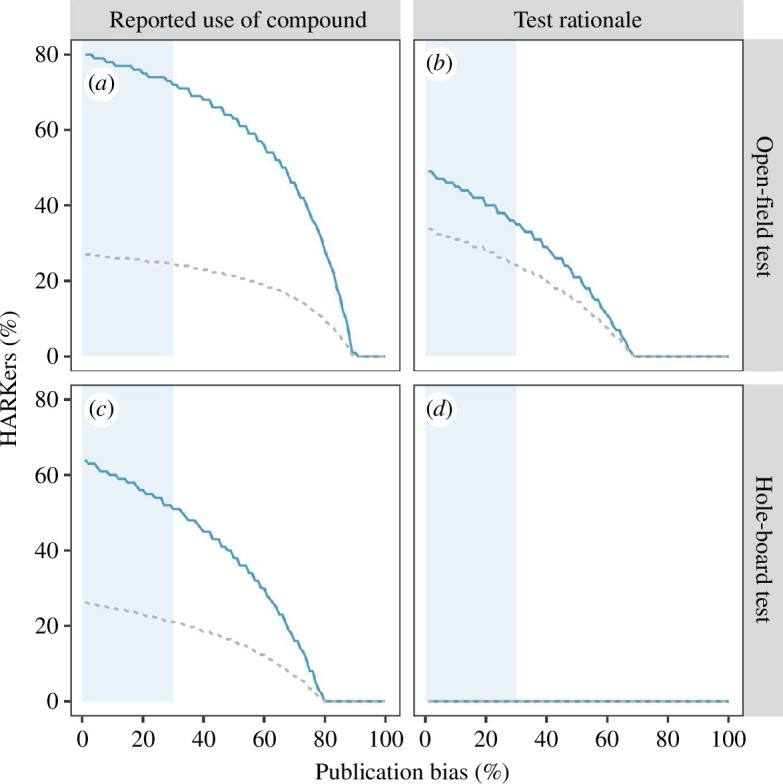
Most likely proportion of HARKing researchers (y-axis) given the percentage of publication bias (x-axis). The shaded area indicates the 95% CI of an estimated publication bias across disciplines as reported in a meta-analysis by Mathur et al [8]. The blue line indicated the estimated proportion of HARKers. The grey dashed line indicated the predicted proportion of HARKers based only on the researchers that have expressed unambiguous rationale.

For the predicted effect of diazepam, figure 3*a* suggests that in the absence of publication bias, the observed reporting pattern for numbers of squares crossed in the open-field test is best described if we assume that 80% of researchers, who made a statement about the predicted effect, were HARKing. Yet, publication bias is a common place [9–11]. A recent meta-analysis summarizing 63 meta-analyses and comprising 12 494 estimates across disciplines suggests an overall publication bias of 15% [8]. Assuming a publication bias of e.g. 30%, which would be within the 95% confidence interval reported by Mathur and VanderWeele, the pattern of observed and predicted effects of diazepam would still suggest 72% of HARKing among those papers finally published. For the number of head dips in the hole-board test, reporting patterns suggest a prevalence of HARKing of 64% in the absence of bias, and still 51% with 30% publication bias (figure 3*c*). Similarly, for the rationale of the use of the open-field test, we found that—in the absence of publication bias—a prevalence of 49% HARKing and—for a 30% publication bias—35% HARKing would be the most likely explanation for the observed reporting pattern (figure 3*b*). For the rationale given for the use of the hole-board test, we found no evidence of HARKing. A post hoc analysis, where the dosage of diazepam was restricted to exactly 1 mg, shows very similar percentages for both tests (electronic supplementary material, figure S1).

## 4. Discussion

We identify a severe problem with current standards of conducting and reporting animal studies. Both the rationale given for using the test, whether to detect anxiolytic or sedative effects, and the predicted effect of diazepam, anxiolytic or sedative, were strongly correlated with the reported test results. The ambiguity in the test rationale and drug effect together with the flexibility in the interpretation of the drug effects seem to hinder critical evaluation of the validity and replicability of these test measures. If all researchers had agreed that the number of squares crossed in the open-field test is a measure of anxiety, then the authors of 66 out of 151 studies would have reported that diazepam did not have an anxiolytic effect, instigating a discussion about the reliability and validity of that test measure. If, on the other hand, all researchers had agreed that the number of squares crossed is simply a measure to detect sedative effects of a compound, the remaining 85 researchers would have reported that diazepam did not have a sedative effect, again questioning the validity of that test measure. However, we found that not in a single one out of the 206 studies, authors mentioned that the observed effects of diazepam on the respective test measure were against their expectations.

We were surprised by this very strong dependency between the postulated effect of the compound or the rationale given for the use of the test and the observed outcome. However, there might be two explanations for this high prevalence of HARKing. First, diazepam is, indeed, a compound where both effects are well known—low dosages of diazepam are supposed to have an anxiolytic effect, while high dosages have a clear sedative effect [12]. This Janus-faced nature of diazepam might make it specifically prone to conscious or subconscious post hoc reasoning. Second, diazepam was usually not the primary compound of interest but added as a positive control to a study investigating a different compound. In such a case, HARKing would not lead to an invalid claim regarding the main outcome (the effect of the novel compound) and, therefore, researchers often might not give as much heed to the interpretation of its effect. Nevertheless, this is problematic, because if the positive control group show unexpected results, this might indicate issues with the validity or robustness of the test paradigm and should be considered a red flag, instigating closer inspection. HARKing with respect to positive controls effectively undermines their control function. Thus, even if HARKing does in this case not affect the primary outcome of interest, it still constitutes a serious problem.

Furthermore, we note that the prevalence of HARKing, expressed as percentage of affected publications, is given for the subpopulation of publications, where authors made unambiguous statements about their expectation regarding the effect and why they made the test. A considerable number of authors made no (or no unambiguous) statements. In those cases, we do not know whether these researchers had clear expectations regarding the outcomes or whether they interpreted them post hoc or not at all. Our best guess might, therefore, be to infer the population-wide prevalence of HARKing from those publications, where explicit statements were made. However, giving those researchers who made no explicit predictions the benefit of the doubt, we could also make the (very optimistic) assumption, that none of them would be HARKing. In this best-case scenario, the overall prevalence of HARKing would be consequently lower (indicated by the grey dashed lines in figure 3)—yet still about 21–34%.

HARKing represents a major threat to a hypothesis-testing framework, where studies are designed to falsify (null) hypotheses [7]. As a form of analytical flexibility, it undermines the robustness and reproducibility of published research for several reasons [13,14]. It hinders the process of falsification of false hypotheses while inflating type I errors, that is, false-positive results mistaken for true effects. It generates a bias in the published literature, missing null results [15,16], and it conceals inconclusive findings, thereby preventing researchers from avoiding such methods in the future. HARKing can distorts the evidence published in the scientific literature, compromising evidence synthesis, and scientific and medical progress; however, a case has been made that HARKing may not always be detrimental to science but only under specific circumstances [17,18].

Besides scientific and economic costs, HARKing may have additional ethical costs, if animals are used for inconclusive research. A potential solution to prevent HARKing is the preregistration of study protocols, where researchers define the hypothesis and analysis plan before a study is performed [13,19]. Such practice compels scientists to keep with their predictions and allows for transparent reporting of what ‘worked’ in a study and what did not. Preregistration and more transparent reporting can aid identification of valid and reproducible test measures, advance self-correction in scientific research and promote responsible research.

## Data Availability

The data that support the findings of this study are openly available on the Open Science Framework repository at [20].
